# Recruitment and retention in obesity prevention and treatment trials targeting minority or low-income children: a review of the clinical trials registration database

**DOI:** 10.1186/s13063-015-1089-z

**Published:** 2015-12-10

**Authors:** Zhaohui Cui, Elisabeth M. Seburg, Nancy E. Sherwood, Myles S. Faith, Dianne S. Ward

**Affiliations:** Department of Nutrition, Gillings School of Global Public Health, University of North Carolina at Chapel Hill, 2202 McGavran-Greenberg Hall, Campus Box 7461, Chapel Hill, NC 27599 USA; HealthPartners Institute for Education and Research, Minneapolis, MN USA

**Keywords:** African American, behavior, children, Hispanic, intervention, lifestyle, low income, recruitment, retention, systematic review

## Abstract

**Background:**

Efforts to recruit and retain participants in clinical trials are challenging, especially in studies that include minority or low-income children. To date, no studies have systematically examined recruitment and retention strategies and their effectiveness in working successfully with this population. We examined strategies employed to recruit or retain minority or low-income children in trials that included an obesity-related behavior modification component.

**Methods:**

First, completed home-, community-, and school-based trials involving minority or low-income children aged 2–17 years were identified in a search of the ClinicalTrials.gov registry. Second, a PubMed search of identified trials was conducted to locate publications pertinent to identified trials. Recruitment and retention rates were calculated for studies that included relevant information.

**Results:**

Our final analytic sample included 43 studies. Of these, 25 studies reported recruitment or retention strategies, with the amount of information varying from a single comment to several pages; 4 published no specific information on recruitment or retention; and 14 had no publications listed in PubMed. The vast majority (92 %) of the 25 studies reported retention rates of, on average, 86 %. Retention rates were lower in studies that: targeted solely Hispanics or African Americans (vs. mixed races of African Americans, whites, and others); involved children and parents (vs. children only); focused on overweight or obese children (vs. general children), lasted ≥1 year (vs. <1 year), were home or community-based (vs. school-based), included nutrition and physical activity intervention (vs. either intervention alone), had body mass index or other anthropometrics as primary outcome measures (vs. obesity-related behavior, insulin sensitivity, etc.). Retention rates did not vary based on child age, number of intervention sessions, or sample size.

**Conclusions:**

Variable amounts of information were provided on recruitment and retention strategies in obesity-related trials involving minority or low-income children. Although reported retention rates were fairly high, a lack of reporting limited the available information. More and consistent reporting and systematic cataloging of recruitment and retention methods are needed. In addition, qualitative and quantitative studies to inform evidence-based decisions in the selection of effective recruitment and retention strategies for trials including minority or low-income children are warranted.

## Background

Successful recruitment and retention are critical for evaluating intervention effectiveness in clinical trials that address childhood obesity. However, the recruitment and retention of participants is challenging, especially in clinical trials that involve ethnic minority or low-income populations in the prevention or treatment of childhood obesity. Problems in participant recruitment may lead to untimely delays in implementation, added financial burden, and failure to meet recruitment goals. Once participants have been recruited, maintaining their engagement across the course of the trial requires thoughtful planning, careful monitoring, and sometimes extraordinary efforts.

Recently, the National Heart, Lung, and Blood Institute convened a workshop to address recruitment and retention strategies in phase 3 and 4 clinical trials. In an article about this initiative, Probstfield and Frye [1] summarized critical steps that must be taken to ensure adequate participant enrollment and retention. These authors noted that trials that involve women and minority populations are more challenging and costly because of issues related to transportation, childcare, and individual and community acceptance. Moreover, reaching minority participants creates additional challenges.

Childhood obesity studies, both for prevention and treatment, present additional challenges related to participant recruitment and retention. Parents and caregivers are often not interested in or have little concern for obesity as a problem and may not recognize excess body weight, especially when it occurs in younger children [2, 3]. An added component of research involving children is that family participation, either direct or indirect, is required. Even when parents or other primary caregivers are not targeted as study participants, family members must provide consent, support, and coordination for the child’s participation in the research study. Thus, recruitment and retention of participants must consider the index child and a parent or guardian for study success.

Childhood obesity intervention trials, especially those conducted within community settings, offer great challenges for participant recruitment and retention because of the time required for baseline measures, intervention delivery, post-intervention testing, and measures of sustainability. Although successful recruitment and retention strategies have been generally described in studies focusing on adults [4] and children [3], no prior reviews have systematically assessed the recruitment and retention of minority or low-income children and families in obesity treatment and prevention studies. In addition, no studies have attempted to determine what information about recruitment and retention is provided in childhood obesity intervention studies following their completion. More information is needed about successful recruitment and retention strategies for interventions that involve minority or low-income children and families to provide researchers with needed information for better design and budgeting for research studies.

The United States Clinical Trials Registration Database (CTRD) offers an excellent study frame to address these issues. For this database, a clinical trial is defined as any research study that assigns human participants to interventions (e.g., a medical product, behavior, or procedure) to evaluate the effects on health outcomes [5]. In 2000, the United States CTRD (ClinicalTrials.gov) was established as an official web platform and catalog for registering a clinical trial. Run by the United States National Library of Medicine, ClinicalTrials.gov was the first online registry for clinical trials and is the largest and most widely used trial registry today. Part of the purpose of the CTRD is to make clinical trial information more widely available and to standardize information provided about trials. In 2005, the International Committee of Medical Journal Editors initiated the policy that trials will be considered for publication only if they were registered before submission [6]. This policy has been followed by a large number of journals [7]. The CTRD is accepted by the International Committee of Medical Journal Editors [6].

Because of the importance of recruitment and retention strategies, the increased participation of community intervention trials in the CTRD, and the provision of information on the trials’ process, a review of the recruitment and retention strategies of childhood obesity prevention and treatment intervention studies located within the database was undertaken. The purpose of the review was to glean collective information from the registered trials, which could be used to improve subsequent childhood obesity interventions and to enhance future recruitment and retention efforts. Specifically, this review aimed to (1) describe strategies employed to recruit minority participants to intervention trials targeting child diet, physical activity, or obesity-related outcomes and assess the success of these recruitment efforts; and (2) examine strategies used to retain participants in these intervention trials and evaluate retention success.

## Methods

The CTRD was searched to identify ‘completed’ trials (as defined by CTRD) that contained information about recruitment and retention of child or adolescent participants in studies with diet, physical activity, or obesity-related outcomes on 6 March 2014. We included home-, community-, and school-based interventions with a behavioral intervention component. Inclusion criteria included: (a) ethnic minority or low-income children or adolescents as the intervention target; (b) diet, physical activity, or obesity-related outcome; (c) a completed trial; and (d) specific information on recruitment or retention numbers and strategies used. Studies were excluded if they tested a specialized diet, medication, dietary supplement, or monitoring device; studied infants (i.e., <2 years of age); or focused on an infectious disease outcome or illness other than obesity or diabetes.

Using the CTRD search engine, specific search terms used included: (underserved OR ‘hidden population’ OR uninsured OR minority OR low income OR Latino OR Latina OR Hispanic OR black OR African American OR Mexican American OR poverty OR vulnerable OR ethnic). Also within the CTRD search engine: the ‘Recruitment’ parameter was constrained to be ‘completed’; the ‘Study type’ parameter was constrained to ‘interventional studies’; the ‘Conditions’ parameter was constrained to (type 2 diabetes OR diabetes mellitus OR obesity OR overweight OR diet OR nutrition OR physical activity OR sedentary behavior); and the ‘Age group’ parameter was constrained to ‘Child (birth to 17 years)’.

As secondary sources of information on recruitment and retention, we searched within CTRD for pertinent papers associated with each identified study. In addition, a PubMed search was conducted using the following information: (CTRD number OR grant number OR intervention name noted in the CTRD) AND name of the principal investigator AND date of study start. All searches of the CTRD and PubMed were conducted by the first author (ZC) after consulting a university librarian assigned to services exclusively for public health research. The first author (ZC) read all of the registration information in an effort to identify appropriate studies. Studies that provided information on recruitment or retention numbers and strategies were retained. Data extraction was performed independently by two authors using tailored tables, and results were cross-checked for accuracy and completeness. Disagreements between the two authors were discussed and resolved in regular writing group meetings.

## Results

### Analytic sample and sample characteristics

**Fig. 1:**
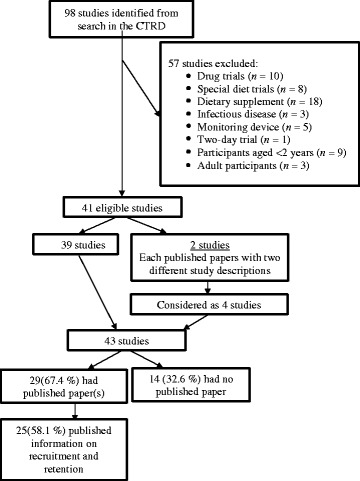
Flowchart for identification of studies and published papers

A total of 98 studies were retrieved from our search of the CTRD (Fig. 1). Of these, 57 studies were excluded for the following reasons: drug trials (*n* = 10); special diet trials (*n* = 8); dietary supplement (*n* = 18); infectious disease (*n* = 3); monitoring device (*n* = 5); 2-day trial (*n* = 1); participants younger than 2 years (*n* = 9) or older than 17 years (*n* = 3). This yielded a total of 41 eligible studies. Search methods identified two additional papers that described studies that were linked to two of the 41 CTRD numbers but appeared to represent slightly different studies (different sample sizes). These were included as separate studies, bringing our final analytic sample total to 43 studies. Of these 43 studies, 29 had at least one published article in a peer-reviewed journal, with 25 having specific information on recruitment or retention of participants. One of the 25 studies (i.e., Girls Health Enrichment Multi-Site Studies or GEMS) included several articles published, from seven different study phases or sites.

Characteristics of the 25 studies included in this review are described in Table 1. More than half of the studies were randomized controlled trials (*n* = 14); five were cluster randomized controlled trials; two were non-randomized controlled trials; and four were trials without a control group. Studies were conducted in various settings, including home or community, including county extension offices, YMCA and childcare centers (*n* = 11), schools (*n* = 7), clinics (*n* = 5), laboratories (*n* = 3). Categories are not mutually exclusive, as some studies had more than one setting. By design, all studies enrolled Hispanics or African Americans, but could have enrolled white participants. Eighty percent of the studies targeted both children and parents. More than 75 % of studies included both nutrition and physical activity intervention components. Two-thirds of the studies lasted less than 1 year. Most studies utilized body mass index (BMI, *n* = 11) or insulin sensitivity or blood glucose metabolism (*n* = 10) as the primary outcome measures, while others used physical activity or fitness (*n* = 5), body fat (*n* = 4), diet (*n* = 3) or adherence behaviors (*n* = 3).

**Table 1:** Characteristics of extracted studies

Reference and CTRD number	Participants				Intervention			Primary outcome
	Child’s race or ethnicity	Child’s body weight status	Child’s age in years (sex)^a^	Parental participation	Setting	Focus	Length	
Hasson *et al.* [14]	Black	Obese	15.4 ± 1.1	Yes	Laboratory	Nutrition, physical activity	16 weeks	Adiposity, inflammation, insulin sensitivity
NCT01441323								
Davis *et al.* [15]	Hispanic	Overweight or obese	14–18	Yes	Laboratory	Nutrition, physical activity	16 weeks	Adiposity, insulin sensitivity
Ventura *et al.* [16]								
NCT00697580								
Azevedo *et al.* [17]	Hispanic	All weights	7–11	Yes	Not reported for dance; at home for TV time	Nutrition, physical activity	2 years	BMI
NCT00476775								
Berry *et al.* [18–20]^b^	Black (63 %), white (32 %), other (5 %)	Overweight or obese	7–10	Overweight or obese	School	Nutrition, physical activity	12 months	Child’s BMI percentile, parent BMI
NCT01378806								
Elizondo-Montemayor *et al.* [21] ^c^	Hispanic	Overweight or obese	6–12	Yes	School	Nutrition	1 school year	BMI percentile, dietary intake and eating habits
NCT01925976								
Wang *et al.* [22, 23] ^b^	Black	All weights	5–7th grade	No	School	Nutrition, physical activity		Feasibility of intervention
NCT00061165								
Black *et al.* [24, 25]	Black	All weights	11–16	Yes	Home and community	Nutrition, physical activity	11 months	BMI
Hurley *et al.* [26]								
Witherspoon *et al.* [27]								
NCT00746083								
Weigensberg *et al.* [28]	Hispanic	Obese	14–17	No	Not clear	Nutrition, physical activity, interactive guided imagery	12 weeks	Insulin sensitivity
NCT01895595								
Wilson *et al.* 2011 [29–31]^b^	Black (73 %), other	All weights	6th grade	No	School	Physical activity	17 weeks	Moderate-to-vigorous physical activity
NCT01028144								
Naar-King *et al.* [32]	Black	Obese	12–17	Yes	Home	Nutrition, physical activity	6 months	BMI, overweight (%), percentage body fat
NCT00604981								
Ritchie *et al.* [33]	Black	Overweight	9–10	Yes	YMCA	Nutrition, physical activity	4–9 seasons	Insulin sensitivity
Sharma *et al.* [34]^d^								
NCT01039116								
Eisenmann *et al.* [35]^d^	Hispanic or black	All weights	3rd–5th grade	Yes	School and community	Nutrition, physical activity	2 years	Physical activity, healthy eating index
NCT01385046								
Barkin *et al.* [36]	Hispanic	All weights	2–6	Yes	Community recreation center	Nutrition, physical activity	12 weeks	BMI
NCT00808431								
Burnet *et al.* [37]^e^	Black	Overweight or obese	9*-*12, with family history of type 2 diabetes mellitus	Yes	Community	Nutrition, physical activity	1 year	Child’s BMI *z* score, parent’s BMI, glucose tolerance
NCT00723853								
Davis *et al.* [38–40]	White (58 %), black (39 %), Hispanic (3 %)	Overweight or obese	7–11	No	Laboratory	Nutrition, physical activity	10–15 weeks	Risk of type 2 diabetes mellitus, VO_2_ max, percentage body fat, visceral fat
Tkacz *et al.* [41]								
Petty *et al.* [42]								
NCT00108901								
Madsen *et al.* [43] ^b^	Hispanic (42 %), Asian (32 %), black (12 %), white (0.6 %), other (13.4 %)	All weights	4th or 5th grade	No	School	Physical activity	24 weeks	Change in minutes of after-school moderate-to-vigorous physical activity, VO_2_ max, BMI
NCT01156103								
Wickham *et al.* [44]	Black (70.3 %), white (26.1 %), Hispanic (1.8 %)	Obese	11–18	Yes	Weight management clinic	Nutrition, physical activity	2 years (results at 6 months reported)	BMI, metabolic indicators, fitness
NCT00167830								
Bean *et al.* [45] ^e^	Black (75.3 %), white (22.0 %), other (2.7 %)	Obese	11–18	Yes	Weight management clinic	Nutrition, physical activity	2 years (results at 6 months reported)	Dietary changes
NCT00167830								
Wysocki *et al.* [46, 47]	White (78.2 %), black (21.0 %), Hispanic (0.8 %)	All weights	12–16.75 with type 1 diabetes mellitus	Yes	Treatment center	Parent–adolescent conflict	12 months (results at 3 months reported)	Family relationships, psychological adjustment to diabetes treatment, treatment adherence, diabetic control
NCT00358059								
Wysocki *et al.* [48–50]	White (63.5 %), black (30.8 %), Hispanic (2.9 %), other (2.9 %)	All weights	11–16, with type 1 diabetes mellitus	Yes	Pediatric center	Parent–adolescent conflict	6 months	Family relationships, treatment adherence, HbA1c, health care use
NCT00358059								
Ellis *et al.* [51, 52]	Black (63 %), white (26 %), other (11 %)	All weights	10–17, with type 1 diabetes mellitus	Yes	Home, community	Home-based psychotherapy	Approximately 6 months	Adherence to medical regimen, metabolic control, hospital use
NCT00519935								
Story *et al.* [2]	Black	Phase I: BMI ≥25th or ≥50th percentile;	8–10 (girls)	Overweight or obese	Community center, school, home	Nutrition, physical activity	Phase I: 12 weeks;	Phase I: process measures;
Rochon *et al.* [53]		Phase II: BMI ≥25th percentile but ≤35 kg/m^2^					Phase II: 2 years	Phase II: change in child’s BMI
Kumanyika *et al.* [54, 55]								
Klesges *et al.* [56, 57]								
Robinson *et al.* [58, 59]								
Stockton *et al.* [60]								
NCT00000615								
Natale *et al.* [61] ^b^	Hispanic (60 %), Haitian (15 %), black (12 %), white (2 %), other (11 %)	All weights	2–5	Yes	Childcare center	Nutrition, physical activity	2 years	Child’s BMI
NCT01722032								
Nansel *et al.* [62]	White (75 %), Hispanic (10 %), black (9 %), other (6 %)	All weights	9–14.9, with type 1 diabetes mellitus	Yes	Pediatric endocrinology clinic	Diabetes management behavior	2 years	HbA1c
NCT00273286								
Janicke *et al.* [63]	White (76.1 %), black (9.8 %), Hispanic (8.5 %), biracial (4.2 %)	Overweight or obese	8–14	Yes	County extension office	Nutrition, physical activity	16 weeks	Change in child’s BMI
Follansbee-Junger *et al.* [64]								
Radcliff *et al.* [65]								
NCT00248677								

*BMI* body mass index

^a^Included both sexes if not specified

^b^Cluster randomized clinical trial

^c^Trial without control group

^d^Non-randomized controlled trial

^e^Pre-post study design

### Recruitment rates and strategies

Recruitment information provided in the studies is described in Table 2. Of the 25 studies, 16 (64 %) did not report a recruitment target; 8 (32 %) did not report capture rate expressed as the ratio of participants who were enrolled to potential participants who were screened. When capture rate was included, it ranged from 10 % to 90 %. Eight (32 %) of the 25 studies did not report formative research information on recruitment. Only eight studies reported recruitment durations, which ranged from 2.5 months (enrolled approximately 60 girls) to 3 years (enrolled 235 children). Recruitment was primarily conducted in community, school, and primary care settings. Specific recruitment strategies were reported in only 14 studies, with the amount of information varying from a single comment to several pages. Common recruitment methods were presentations, flyers, brochures, posters, media advertisements, phone calls, and word-of-mouth. Two-thirds of studies did not report any information on barriers for recruitment. When barriers were reported, they included participants’ time constraints, competing demands, transportation safety and distance, childcare needs, lack of interest, and study funding limitations.

**Table 2:** Study recruitment: effectiveness, setting, strategies employed, and barriers reported

Reference	Sample size	Reach (% capture)	Formative research	Recruitment duration	Recruitment setting	Recruitment strategies	Recruitment barriers
Hasson *et al.* [14]	58 families	11.6	Yes	–	–	–	–
Davis *et al.* [15]	68 families	17.0	Yes	–	–	–	–
Ventura *et al.* [16]							
Azevedo *et al.* [17]	252 families	–	–	–	Community	–	–
Berry *et al.* [18–20]	358 parent–child dyads	27.5	Yes	2 years 9 months	School	1) Meeting with school staff	–
						2) Printed study information	
						3) Presentation to children and parents	
						4) Printed study contact information	
						5) Friendly manner	
Elizondo-Montemayor *et al.* [21]	125 caregiver–child dyads	9.6	–	–	School	–	–
Wang *et al.* [22, 23]	249 children	37.1	Yes	–	School	–	–
Black *et al.* [24, 25]	235 children	–	–	1 year 10 months	School	–	–
Hurley *et al.* [26]							
Witherspoon *et al.* [27]							
Weigensberg *et al.* [28]	35 adolescents	62.5	Yes	–	Pediatric clinics, health fairs	–	School vacation
Wilson *et al.* 2011 [29–31]	1422 children	91.0	Yes	–	School and home	1) Presentation to parents and students	–
						2) Home visit	
Naar-King *et al.* [32]	49 families	69.0	Yes	–	An urban adolescent medicine clinic	–	1) Time constraint;
							2) Lack of interest
Ritchie *et al.* [33]	235 families	–	Yes	3 years	School, community	1) Announcements	1) Transportation;
Sharma *et al.* [34]						2) Incentives	2) Competing demands;
							3) Distrust;
Eisenmann *et al.* [35]	434 families	57.0	–	–	School	–	–
Barkin *et al.* [36]	106 parent–child dyads	22.2	–	4–5 months	Cooperating community agencies such as social service agencies, pediatric clinics, community centers	1) Printed study information	1) Transportation;
						2) Radio	2) On-site childcare
						3) Participant referral	
Burnet *et al.* [37]	29 families	–	Yes	–	Community, pediatric clinics	Printed study information	–
Davis *et al.* [38–40]	222 children	26.4 %	–	2 years 8 months	School	Printed study information	–
Tkacz *et al.* [41]							
Petty *et al.* [42]							
Madsen *et al.* [43]	156 children, six schools	11.7 % , 50 %, 89.7 %	Yes	–	School	Presentation to school staff	Change in school administration
Wickham *et al.* [44]	165 adolescents	–	–	2 years 4 months	Comprehensive weight management program	Healthcare provider referral	–
Bean *et al.* [45]	186 adolescents	–	Yes	2 years 11 months	Health care, school, community	Healthcare provider referral	–
Wysocki *et al.* [46, 47]	119 families	31.3 %	Yes	–	–	–	1) Transportation;
							2) Time constraint
Wysocki *et al.* [48–50]	104 families	23.9 %	Yes	–	Pediatric diabetes centers	1) Mailed invitation letter	Funding limitation
						2) Phone call	
Ellis *et al.* [51, 52]	127 adolescents	69.8 %	Yes	–	Endocrinology clinic	–	–
Story *et al.* [2]	Phase I: 35–61 girls;	Phase I : not reported;	Yes	Phase I: 2.5–4 months^a^;	Community churches, community centers, community events and school	1) Active placebo study group	Phase I:
Rochon *et al.* [53]	Phase II: 261–303 girls	Phase II: 48.1 %-65.4 %		Phase II: 17 months		2) Media adverts, stories, interviews	1) No-treatment control group;
Kumanyika *et al.* [54, 55]						3) Flyers to homes	2) Parents interested in both child health and self-esteem programs, while children interested in fun programs;
Klesges *et al.* [56, 57]						4) Presentations to families at community and school	3) Blood draw.
Robinson *et al.* [58, 59]						5) Separate consent for blood draw, which was not required for participation	Phase II:
Stockton *et al.* [60]							1) School vacation
							2) Study staff issues
							3) Study site locations
Natale *et al.* [61]	1105 children	–	–	–	Child care center	–	–
Nansel *et al.* [62]	390 families	69.1 %	–		Pediatric endocrinology clinics	–	–
Janicke *et al.* [63]	93 parent–child dyads	83.8 %	Yes		Community and school	1) Printed study information	–
Follansbee-Junger *et al.* [64]						2) Community presentations	
Radcliff *et al.* [65]						3) Toll-free line	

^a^11.7 % of screened schools, 50 % of eligible schools at principals’ meeting, 89.7 % of children

### Retention rates and strategies

Table 3 shows the average retention rates from individual studies based on study characteristics. Of the 25 studies examined, 23 studies reported retention rates, with an average rate of 86 %. Studies solely targeting Hispanics or African Americans had lower average retention rates, of 82.8 % and 83.5 %, respectively, than those targeting both ethnic minority and white participants (92.1 %). Three studies included children only; the average retention rate from these studies was higher than the average retention rate from studies that involved both children and parents (91.1 % vs. 85.6 %). On average, studies that focused on overweight or obese children had lower retention rates than those that targeted children generally (79.6 % vs. 90.0 %). Accordingly, treatment studies had a lower average retention rate than prevention studies, especially when the intervention lasted over 1 year (74.0 % vs. 88.8 %). Overall, longer-term studies produced lower retention rates than shorter-term studies, especially for treatment studies (74.0 % for ≥ 1 year vs. 87.2 % for < 1 year). Interestingly, studies with BMI or anthropometrics as primary outcome measures had lower retention rates than studies with other primary outcome measures (e.g., obesity-related behavior, insulin sensitivity; 82.9 % vs. 89.0 %). Home- or community-based studies had lower retention rates than school-based studies (85.5 % vs. 91.7 %). Studies including both nutrition and physical activity intervention components tended to have lower retention rates than studies focusing solely on nutrition or physical activity (85.0 % vs. 92.8 %). Retention rates did not differ by the mean age of children (<12 years vs. ≥ 12 years), number of intervention sessions (≤12 vs. ≥13), or study sample size (<100 vs. ≥100).

**Table 3:** Average retention rates by study characteristics

	Number of studies	Study enrollment^a^	Study retention^b^	Average retention rates
Race or ethnicity				
Hispanic	5	586	511	82.8
African American	10	1331	1059	83.5
African American, white and other	8	1927	1763	92.1
Intervention target				
Children	3	413	388	91.1
Children and parent	20	3431	2945	85.6
Body weight status				
Overweight or obese	9	1581	1314	79.6
All weights	10	1523	1334	90.0
Body weight status not measured	4	740	685	92.6
Study type				
Prevention	10	1523	1334	90.0
Treatment	13	2321	1999	83.6
Intervention length				
<1 year	16	1658	1461	88.6
≥1 year	7	2186	1872	81.1
Study type and treatment length				
Prevention <1 year	7	707	614	90.4
Prevention ≥1 year	3	816	720	88.8
Treatment <1 year	9	951	847	87.2
Treatment ≥1 year	4	1073	873	74.0
Primary outcome				
BMI or anthropometrics	10	2342	2026	82.9
Other (behavior, physiology, etc.)	13	1502	1307	89.0
Intervention setting^c^				
School	5	1273	1151	91.7
Home or community	15	2410	2051	85.5
Laboratory	2	126	102	81.1
Main intervention group				
Nutrition or physical activity	4	755	712	92.8
Nutrition and physical activity	19	3089	2621	85.0
Study design				
Randomized controlled trial	19	2739	2440	89.3
Cluster randomized controlled trial	2	745	656	75.6
Controlled trial	1	235	136	57.9
Trial without control	1	125	101	80.8
Mean age of children^d^				
<12 years	15	2708	2333	86.2
≥12 years	8	1136	1000	86.7
Number of intervention sessions^e^				
≤12	7	752	636	86.3
≥13	15	2840	2445	85.5
Sample size				
<100	9	493	435	86.9
≥100	14	3351	2898	86.0

^a^The sum of numbers of participants enrolled in individual studies

^b^The sum of numbers of participants retained in individual studies

^c^Intervention setting was not reported in the study by Weigensberg *et al.* [28]

^d^<12 years group includes one study with participants aged 8–14 years; ≥12 years group includes one study with participants aged 9–14 years, one study with participants aged 10–17 years and two studies with participants aged 11–16 years

^e^Number of intervention sessions was not reported in the study by Azevedo *et al.* [17]

Of the 25 studies, 18 (72 %) reported retention strategies. We analyzed and coded retention strategies used in these studies and categorized strategies into intervention design, incentive, project bond, participant convenience, and participant tracking (Table 4). Retention strategies related to intervention design included culturally appropriate intervention activities and staff, developmentally appropriate goals for participants, a run-in phase before randomization, provision of counseling or technical support to help participants address participation barriers, regular interventionist–principal investigator meetings to ensure participant-centered intervention, and the use of a delayed or alternative intervention for control group. Incentives, such as grocery gift cards, gifts, cash, food, recipe books, and exercise equipment, were offered for intervention attendance or completion at each data collection point. Study staff also established project bonds with participants or the broader community by building staff–participant relationships, and regular communication with participants, such as thank-you notes, postcards, or project newsletters. Retention strategies related to participant convenience included transportation support to and from intervention activities or data collection, make-up sessions for missed intervention sessions, upcoming event reminders, childcare services, and optional days or home visits for data collection. To facilitate tracking participants, complete contact information was collected from participants at baseline and a tracking database established. One study mentioned sending personalized letters to participants who were difficult to reach, to schedule data collection appointments. Common retention methods used were alternative or delayed interventions for the control groups, monetary incentives, regular contact and relationship building with participants and the community, provision of transportation support, and offering flexible intervention and measurement visits.

**Table 4:** Retention strategies described in articles reviewed

Reference	Retention strategy					Retention rate
	Intervention design	Incentive	Project bond	Participant convenience	Participant tracking	
Davis *et al.* [15]	Run-in phase	Weekly grocery gift cards	–	Transportation support	–	79.4 % (54/68)
Ventura *et al.* [16]						
Azevedo *et al.* [17]	–	Rewards for retention	–	–	–	100 % (252/252)
Berry *et al.* [18–20]	1) Delayed intervention for control group 2) Counseling or support	1) Exercise equipment 2) Money for data collection 3) Food 4) Gifts	1) Regular contact 2) Refrigerator magnet 3) Building staff–participant relationship	1) Reminder message 2) Flexible data collection days 3) Childcare 4) Transportation support	1) Complete contact information 2) Toll-free line 3) Tracking letter	89.1 % (638/716)
Elizondo-Montemayor *et al.* [21]	–	–	Building staff–participant relationship	Reminder message	–	80.8 % (101/125)
Black *et al.* [24, 25]	Culturally sensitive	–	–	–	–	78.3 % (184/235)
Hurley *et al.* [26]						
Witherspoon *et al.* [27]						
Weigensberg *et al.* [28]		–	–	Transportation support Make-up session	–	82.9 % (29/35)
Ritchie *et al.* [33]	1) Alternative intervention for control group 2) Counseling or support 3) Culturally sensitive	1) Exercise equipment 2) Recipe books	1) Building staff–participant relationship 2) Regular contact	Transportation support	–	57.9 % (136/235)
Sharma *et al.* [34]						
Burnet *et al.* [37]	1) Culturally sensitive 2) Activities at YMCA and grocery stores	–	Building staff–participant relationship	1) Convenient intervention sites 2) Transportation support 3) Child care	–	62.1 % (18/29)
Davis *et al.* [38–40]	–	1) Weekly prizes 2) Increasing money for data collections 3) Food at intervention session	Regular contact	Transportation support	–	94.1 % (209/222)
Tkacz *et al.* [41]						
Petty *et al.* [42]						
Wickham *et al.* [44]	–	YMCA membership	–	–	–	–
Bean *et al.* [45]	–	1) YMCA membership 2) Grocery store gift card for data collection	–	–	–	–
Wysocki *et al.* [46, 47]	Alternative intervention for control group	1) Money for each data collection 2) Money for completing all intervention sessions	–	–	–	96.6 % (115/119)
Wysocki *et al.* [48–50]	Alternative intervention for control group	1) Money for each data collection 2) Money for completion of all intervention sessions	–	–	–	88.5 % (92/104)
Ellis *et al.* [51, 52]	Alternative intervention for control group	–	–	Convenient intervention sites	–	92.9 % (118/127)
Story *et al.* [2]	1) Alternative intervention for control group 2) Fun intervention activities 3) Culturally sensitive	1) Gift for intervention attendance 2) Money 3) Increasing money for data collections 4) Additional money for blood draw 5) Food	1) Family nights 2) Regular contact 3) Build relationship between study and broader community	1) Convenient intervention sites 2) Flexible study procedures and measurement visits 3) Home visits for data collection 4) Transportation support 5) Childcare 6) Email and telephone reminders	1) Complete contact information 2) Tracking database 3) Calls from ‘non-identifiable’ cell phones	Phase I:
Rochon *et al.* [53]						ᅟ
Kumanyika *et al.* [54, 55]						91.4 % (32/35) and 100 % (60/60)
Klesges *et al.* [56, 57]						Phase II:
Robinson *et al.* [58, 59]						80.2 % (243/303) and 86.2 % (225/261)
Stockton *et al.* [60]						
Natale *et al.* [61]	Alternative intervention for control group	Incentives (not specified)	Regular contact	–	–	–
Nansel *et al.* [62]	Alternative intervention for control group	1) Money for completing all data collections 2) Additional money for child providing blood glucose meter data	1) Appointment reminder calls 2) Follow-up calls after appointment	1) Transportation support 2) Midpoint evaluations by telephone	–	92.3 % (360/390)
Janicke *et al.* [63]	1) Delayed intervention for control group 2) Proper participant goals 3) Person-centered intervention	1) Drawing for gift card at weekly child session 2) Gift card per family for each session 3) Money for data collections 4) Food	1) Build community connections 2) Regular contact 3) Phone calls to participants after missed sessions	Make-up sessions	–	87.1 % (81/93)
Follansbee-Junger *et al.* [64]						
Radcliff *et al.* [65]						

## Discussion

### Summary of key findings

Our systematic review of recruitment and retention of minority or low-income children into obesity-related intervention trials identified 41 completed studies in the CTRD, two of which were linked to two studies. Of these 43 studies, only 25 (60 %) had published information on recruitment or retention in a peer-reviewed article, with considerable variation in the amount of information provided among studies. A further ≈ 10 % included no information about recruitment and retention in their papers. Even when we examined only the studies completed 2 years prior to the close date of our CTRD search, more than 30 % had no publications in peer-review journals. Although most studies with relevant information reported high retention rates, differences in retention rates existed by participant characteristics (i.e., race, obesity status, involving parents or caregivers) and study design (i.e., prevention or treatment, study duration, primary outcome, home-, community-, or school-based).

### Previous studies that have examined recruitment and retention in this population

Two other studies have systematically examined published articles about recruitment and retention of children into obesity-related studies. Schoeppe *et al.* [3] summarized strategies used to recruit and retain children in behavioral health risk factor studies that achieved high capture rates and low attrition rates, while Amon *et al.* [8] systematically reviewed literature that included the use of Facebook to recruit 10–18-year-old children into studies that aimed to address physical or mental health issues. The authors found that paid advertising on Facebook was effective in recruiting these participants. These two studies used published literature only as their study frame; thus, their results did not cover studies without publications and could not evaluate the proportion of studies conducted with published information on recruitment and retention. Furthermore, these reviews focused on youth generally; thus, it is unclear whether findings can be generalized to minority or low-income children.

### Qualitative and quantitative evidence in recruitment and retention

The articles identified in our review mainly provided narrative descriptions of recruitment and retention strategies used, investigators’ opinions on the effectiveness of these strategies, and lessons learned in individual studies. While this describes important qualitative study experiences related to recruitment and retention strategies, quantitative assessments of these strategies may also improve our understanding of their correlates and effects. Two prior observational studies have quantitatively examined factors associated with the success of recruitment and retention in intervention studies. Using discriminant function analysis and analysis of variance, Coatsworth *et al.* [9] found that retention patterns (i.e., non-attenders, variable attenders or consistently high attenders over intervention sessions) were associated with sociodemographic and child- and family-level characteristics in a family-based intervention aiming to prevent substance use in adolescent girls. Another study using chi-square analyses found that attrition of adolescent girls (the majority being African Americans) involved in a randomized controlled trial of a HIV-prevention intervention was associated with recruiters’ experiences, recruitment method, contact status, and parental awareness of study participation [10]. Our study is the first to examine retention rates quantitatively by participant characteristics and study design in obesity-related trials conducted in minority or low-income children and found results as expected.

In addition to retrospective analysis of the recruitment and retention efforts, prospective studies designed to test specific recruitment and retention strategies are needed. The randomized clinical trial design is considered to provide the strongest causal evidence. We identified three randomized trials that examined the effectiveness of direct mail letters containing different information in the recruitment of minority adults. For example, Brown *et al.* [11] randomly assigned 30,000 minority women into four groups formed by a factorial design: ethnically specific or generic statement on disease risk and personalized or non-personalized letterhead. They found that women who received letters with the ethnically specific statements were 34 % more likely to respond than women who received letters with a generic statement, while there was no significant difference in response between women who received personalized letters and those who received non-personalized letters. However, we did not identify any randomized controlled trials that examined the effect of recruitment and retention strategies in minority or low-income children. Considering the limited amount of quantitative evidence available, further analytical study is needed to examine the success rates of recruitment and retention strategies in a broader scope.

### Limited publications available

We found that one-third of eligible studies had not published a peer-reviewed paper. This proportion remained true if we allowed for additional time for manuscripts to reach the publication stage by excluding studies that were completed less than 2 years before our search of the CTRD. Ross *et al.* [12] examined 635 clinical trials funded by the National Institutes of Health and registered within CTRD and found that more than half of the trials did not publish an article in a peer-reviewed journal indexed by Medline within 2.5 years of trial completion. Furthermore, after 51 months of trial completion, a third of trials remained unpublished. Multiple factors might have contributed to this high non-publication rate, including those beyond the control of the investigators [12, 13]. Ross *et al.* [12] also suggested that 12–24 months should be the goal for results from clinical trials to be published. Furthermore, among studies with published peer-reviewed papers, the scope and amount of information reported varied. The non-publication of studies and inconsistent report of recruitment and retention hinders the sharing of experiences and lessons learned, as well as limiting the synthesis of data across studies. Reporting guidelines, including STrengthening the Reporting of OBservational studies in Epidemiology (STROBE) and Consolidated Standards of Reporting Trials (CONSORT), have improved the reporting of observational and experimental studies in journals that support these guidelines. The development of guidelines for reporting recruitment and retention would be a first step in improving the quality of information reported in this area.

### Strengths and limitations

An advantage of our study is that we used the CTRD as the study frame and focused specifically on minority or low-income participants. In addition, the studies included varied substantially in terms of participants’ characteristics and study design, which allowed us to describe recruitment and retention strategies more broadly and to examine the retention rates quantitatively by study characteristics. Our study has limitations. We searched only one trial registry. However, most obesity-related trials conducted in the United States after the launch of the CTRD might have been registered in this database. In addition, the limited number of studies identified in our study hampered our ability to conduct multivariate analysis, to examine factors associated with retention rates.

## Conclusions

In conclusion, although studies with a published, peer-reviewed article generally achieved high retention rates, limited information on recruitment and retention strategies was available. There is a need for more consistent reporting and systematic cataloging of recruitment and retention methods. Both qualitative and quantitative evidence are warranted to inform evidence-based decisions in choosing effective recruitment and retention strategies for trials involving minority or low-income children.

## Abbreviations

BMI: body mass index
CONSORT: Consolidated Standards of Reporting Trials
CTRD: Clinical Trials Registration Database
GEMS: Girls Health Enrichment Multi-Site Studies
STROBE: STrengthening the Reporting of OBservational studies in Epidemiology

## References

[1] Probstfield JL, Frye RL (2011). Strategies for recruitment and retention of participants in clinical trials. JAMA..

[2] Story M, Sherwood NE, Obarzanek E, Beech BM, Baranowski JC, Thompson NS (2003). Recruitment of African-American pre-adolescent girls into an obesity prevention trial: the GEMS pilot studies. Ethn Dis..

[3] Schoeppe S, Oliver M, Badland HM, Burke M, Duncan MJ (2014). Recruitment and retention of children in behavioral health risk factor studies: REACH strategies. Int J Behav Med..

[4] Yancey AK, Ortega AN, Kumanyika SK (2006). Effective recruitment and retention of minority research participants. Annu Rev Public Health..

[5] National Institutes of Health. ClinicalTrials.gov Background. http://www.clinicaltrials.gov/ct2/about-site/background. Accessed 1 Dec 2014.

[6] International Committee of Medical Journal Editors. Clinical Trials Registration. http://www.icmje.org/about-icmje/faqs/clinical-trials-registration/. Accessed 1 Dec 2014.

[7] International Committee of Medical Journal Editors. Journals Following the ICMJE Recommendations. http://www.icmje.org/journals-following-the-icmje-recommendations/. Accessed 1 Dec 2014.

[8] Amon KL, Campbell AJ, Hawke C, Steinbeck K (2014). Facebook as a recruitment tool for adolescent health research: a systematic review. Acad Pediatr..

[9] Coatsworth JD, Duncan LG, Pantin H, Szapocznik J (2006). Retaining ethnic minority parents in a preventive intervention: the quality of group process. J Prim Prev..

[10] Seibold-Simpson S, Morrison-Beedy D (2010). Avoiding early study attrition in adolescent girls: impact of recruitment contextual factors. West J Nurs Res..

[11] Brown SD, Lee K, Schoffman DE, King AC, Crawley LM, Kiernan M (2012). Minority recruitment into clinical trials: experimental findings and practical implications. Contemp Clin Trials..

[12] Ross JS, Tse T, Zarin DA, Xu H, Zhou L, Krumholz HM (2012). Publication of NIH funded trials registered in ClinicalTrials.gov: cross sectional analysis. BMJ.

[13] Hudson KL, Collins FS (2015). Sharing and reporting the results of clinical trials. JAMA.

[14] Hasson RE, Adam TC, Davis JN, Kelly LA, Ventura EE, Byrd-Williams CE (2012). Randomized controlled trial to improve adiposity, inflammation, and insulin resistance in obese African-American and Latino youth. Obesity (Silver Spring).

[15] Davis JN, Kelly LA, Lane CJ, Ventura EE, Byrd-Williams CE, Alexandar KA (2009). Randomized control trial to improve adiposity and insulin resistance in overweight Latino adolescents. Obesity (Silver Spring)..

[16] Ventura E, Davis J, Byrd-Williams C, Alexander K, McClain A, Lane CJ (2009). Reduction in risk factors for type 2 diabetes mellitus in response to a low-sugar, high-fiber dietary intervention in overweight Latino adolescents. Arch Pediatr Adolesc Med..

[17] Azevedo KJ, Mendoza S, Fernandez M, Haydel KF, Fujimoto M, Tirumalai EC (2013). Turn off the TV and dance! Participation in culturally tailored health interventions: implications for obesity prevention among Mexican American girls. Ethn Dis..

[18] Berry DC, Neal M, Hall EG, McMurray RG, Schwartz TA, Skelly AH (2013). Recruitment and retention strategies for a community-based weight management study for multi-ethnic elementary school children and their parents. Public Health Nurs..

[19] Berry DC, Schwartz TA, McMurray RG, Skelly AH, Neal M, Hall EG (2014). The family partners for health study: a cluster randomized controlled trial for child and parent weight management. Nutr Diabetes..

[20] Berry DC, McMurray R, Schwartz TA, Skelly A, Sanchez M, Neal M (2012). Rationale, design, methodology and sample characteristics for the family partners for health study: a cluster randomized controlled study. BMC Public Health.

[21] Elizondo-Montemayor L, Moreno-Sanchez D, Gutierrez NG, Monsivais-Rodriguez F, Martinez U, Lamadrid-Zertuche AC (2014). Individualized tailor-made dietetic intervention program at schools enhances eating behaviors and dietary habits in obese Hispanic children of low socioeconomic status. Sci World J.

[22] Wang Y, Tussing L, Odoms-Young A, Braunschweig C, Flay B, Hedeker D (2006). Obesity prevention in low socioeconomic status urban African-American adolescents: study design and preliminary findings of the HEALTH-KIDS study. Eur J Clin Nutr..

[23] Wang Y, Li J, Caballero B (2009). Resemblance in dietary intakes between urban low-income African-American adolescents and their mothers: the healthy eating and active lifestyles from school to home for kids study. J Am Diet Assoc..

[24] Black MM, Hager ER, Le K, Anliker J, Arteaga SS, Diclemente C (2010). Challenge! Health promotion/obesity prevention mentorship model among urban, black adolescents. Pediatrics..

[25] Black MM, Arteaga SS, Sanders J, Hager ER, Anliker JA, Gittelsohn J (2012). College mentors: a view from the inside of an intervention to promote health behaviors and prevent obesity among low-income, urban, African American adolescents. Health Promot Pract.

[26] Hurley KM, Oberlander SE, Merry BC, Wrobleski MM, Klassen AC, Black MM (2009). The healthy eating index and youth healthy eating index are unique, nonredundant measures of diet quality among low-income, African American adolescents. J Nutr.

[27] Witherspoon D, Latta L, Wang Y, Black MM (2013). Do depression, self-esteem, body-esteem, and eating attitudes vary by BMI among African American adolescents?. J Pediatr Psychol..

[28] Weigensberg MJ, Lane CJ, Avila Q, Konersman K, Ventura E, Adam T (2014). Imagine HEALTH: results from a randomized pilot lifestyle intervention for obese Latino adolescents using Interactive Guided Imagery^SM^. BMC Complement Altern Med.

[29] Wilson DK, Van Horn ML, Kitzman-Ulrich H, Saunders R, Pate R, Lawman HG (2011). Results of the ‘active by choice today’ (ACT) randomized trial for increasing physical activity in low-income and minority adolescents. Health Psychol..

[30] Wilson DK, Kitzman-Ulrich H, Williams JE, Saunders R, Griffin S, Pate R (2008). An overview of ‘the active by choice today’ (ACT) trial for increasing physical activity. Contemp Clin Trials..

[31] Wilson DK, Lawman HG, Segal M, Chappell S (2011). Neighborhood and parental supports for physical activity in minority adolescents. Am J Prev Med..

[32] Naar-King S, Ellis D, Kolmodin K, Cunningham P, Jen KL, Saelens B (2009). A randomized pilot study of multisystemic therapy targeting obesity in African-American adolescents. J Adolesc Health..

[33] Ritchie LD, Sharma S, Ikeda JP, Mitchell RA, Raman A, Green BS (2010). Taking action together: a YMCA-based protocol to prevent type-2 diabetes in high-BMI inner-city African American children. Trials.

[34] Sharma S, Fleming SE (2012). One-year change in energy and macronutrient intakes of overweight and obese inner-city African American children: effect of community-based taking action together type 2 diabetes prevention program. Eat Behav..

[35] Eisenmann JC, Alaimo K, Pfeiffer K, Paek HJ, Carlson JJ, Hayes H (2011). Project FIT: rationale, design and baseline characteristics of a school- and community-based intervention to address physical activity and healthy eating among low-income elementary school children. BMC Public Health.

[36] Barkin SL, Gesell SB, Po’e EK, Escarfuller J, Tempesti T (2012). Culturally tailored, family-centered, behavioral obesity intervention for Latino-American preschool-aged children. Pediatrics..

[37] Burnet DL, Plaut AJ, Wolf SA, Huo D, Solomon MC, Dekayie G (2011). Reach-out: a family-based diabetes prevention program for African American youth. J Natl Med Assoc..

[38] Davis CL, Tomporowski PD, Boyle CA, Waller JL, Miller PH, Naglieri JA (2007). Effects of aerobic exercise on overweight children’s cognitive functioning: a randomized controlled trial. Res Q Exerc Sport..

[39] Davis CL, Tomporowski PD, McDowell JE, Austin BP, Miller PH, Yanasak NE (2011). Exercise improves executive function and achievement and alters brain activation in overweight children: a randomized, controlled trial. Health Psychol..

[40] Davis CL, Pollock NK, Waller JL, Allison JD, Dennis BA, Bassali R (2012). Exercise dose and diabetes risk in overweight and obese children: a randomized controlled trial. JAMA..

[41] Tkacz J, Young-Hyman D, Boyle CA, Davis CL (2008). Aerobic exercise program reduces anger expression among overweight children. Pediatr Exerc Sci..

[42] Petty KH, Davis CL, Tkacz J, Young-Hyman D, Waller JL (2009). Exercise effects on depressive symptoms and self-worth in overweight children: a randomized controlled trial. J Pediatr Psychol..

[43] Madsen K, Thompson H, Adkins A, Crawford Y (2013). School-community partnerships: a cluster-randomized trial of an after-school soccer program. JAMA Pediatr..

[44] Wickham EP, Stern M, Evans RK, Bryan DL, Moskowitz WB, Clore JN (2009). Prevalence of the metabolic syndrome among obese adolescents enrolled in a multidisciplinary weight management program: clinical correlates and response to treatment. Metab Syndr Relat Disord..

[45] Bean MK, Mazzeo SE, Stern M, Evans RK, Bryan D, Ning Y (2011). Six-month dietary changes in ethnically diverse, obese adolescents participating in a multidisciplinary weight management program. Clin Pediatr (Phila).

[46] Wysocki T, Harris MA, Greco P, Bubb J, Danda CE, Harvey LM (2000). Randomized, controlled trial of behavior therapy for families of adolescents with insulin-dependent diabetes mellitus. J Pediatr Psychol..

[47] Wysocki T, Greco P, Harris MA, Bubb J, White NH (2001). Behavior therapy for families of adolescents with diabetes: maintenance of treatment effects. Diabetes Care..

[48] Wysocki T, Harris MA, Buckloh LM, Mertlich D, Lochrie AS, Taylor A (2006). Effects of behavioral family systems therapy for diabetes on adolescents’ family relationships, treatment adherence, and metabolic control. J Pediatr Psychol..

[49] Wysocki T, Harris MA, Buckloh LM, Mertlich D, Lochrie AS, Mauras N (2007). Randomized trial of behavioral family systems therapy for diabetes: maintenance of effects on diabetes outcomes in adolescents. Diabetes Care..

[50] Wysocki T, Harris MA, Buckloh LM, Mertlich D, Lochrie AS, Taylor A (2008). Randomized, controlled trial of behavioral family systems therapy for diabetes: maintenance and generalization of effects on parent-adolescent communication. Behav Ther..

[51] Ellis DA, Frey MA, Naar-King S, Templin T, Cunningham P, Cakan N (2005). Use of multisystemic therapy to improve regimen adherence among adolescents with type 1 diabetes in chronic poor metabolic control: a randomized controlled trial. Diabetes Care..

[52] Ellis D, Naar-King S, Templin T, Frey M, Cunningham P, Sheidow A (2008). Multisystemic therapy for adolescents with poorly controlled type 1 diabetes: reduced diabetic ketoacidosis admissions and related costs over 24 months. Diabetes Care..

[53] Rochon J, Klesges RC, Story M, Robinson TN, Baranowski T, Obarzanek E (2003). Common design elements of the Girls Health Enrichment Multi-site Studies (GEMS). EthnDis.

[54] Kumanyika SK, Story M, Beech BM, Sherwood NE, Baranowski JC, Powell TM (2003). Collaborative planning for formative assessment and cultural appropriateness in the girls health enrichment multi-site studies (GEMS): a retrospection. Ethn Dis..

[55] Kumanyika S, Fassbender J, Phipps E, Tan-Torres S, Localio R, Morales KH (2011). Design, recruitment and start up of a primary care weight loss trial targeting African American and Hispanic adults. Contemp Clin Trials..

[56] Klesges RC, Obarzanek E, Klesges LM, Stockton MB, Beech BM, Murray DM (2008). Memphis girls health enrichment multi-site studies (GEMS). Phase 2: design and baseline. Contemp Clin Trials.

[57] Klesges RC, Obarzanek E, Kumanyika S, Murray DM, Klesges LM, Relyea GE (2010). The Memphis girls’ health enrichment multi-site studies (GEMS): an evaluation of the efficacy of a 2-year obesity prevention program in African American girls. Arch Pediatr Adolesc Med..

[58] Robinson TN, Matheson DM, Kraemer HC, Wilson DM, Obarzanek E, Thompson NS (2010). A randomized controlled trial of culturally tailored dance and reducing screen time to prevent weight gain in low-income African American girls: Stanford GEMS. Arch Pediatr Adolesc Med..

[59] Robinson TN, Kraemer HC, Matheson DM, Obarzanek E, Wilson DM, Haskell WL (2008). Stanford GEMS phase 2 obesity prevention trial for low-income African-American girls: design and sample baseline characteristics. Contemp Clin Trials..

[60] Stockton MB, McClanahan BS, Lanctot JQ, Klesges RC, Beech BM (2012). Identification of facilitators and barriers to participation in weight gain prevention research by African American girls. Contemp Clin Trials..

[61] Natale R, Scott SH, Messiah SE, Schrack MM, Uhlhorn SB, Delamater A (2013). Design and methods for evaluating an early childhood obesity prevention program in the childcare center setting. BMC Public Health.

[62] Nansel TR, Iannotti RJ, Liu A (2012). Clinic-integrated behavioral intervention for families of youth with type 1 diabetes: randomized clinical trial. Pediatrics..

[63] Janicke DM, Sallinen BJ, Perri MG, Lutes LD, Huerta M, Silverstein JH (2008). Comparison of parent-only vs family-based interventions for overweight children in underserved rural settings: outcomes from project STORY. Arch Pediatr Adolesc Med..

[64] Follansbee-Junger K, Janicke DM, Sallinen BJ (2010). The influence of a behavioral weight management program on disordered eating attitudes and behaviors in children with overweight. J Am Diet Assoc..

[65] Radcliff TA, Bobroff LB, Lutes LD, Durning PE, Daniels MJ, Limacher MC (2012). Comparing costs of telephone vs face-to-face extended-care programs for the management of obesity in rural settings. J Acad Nutr Diet..

